# Does training on performance based financing make a difference in performance and quality of health care delivery? Health care provider’s perspective in Rungwe Tanzania

**DOI:** 10.1186/1472-6963-14-154

**Published:** 2014-04-04

**Authors:** Rachel Manongi, Declare Mushi, Joachim Kessy, Saria Salome, Bernard Njau

**Affiliations:** 1 Kilimanjaro Christian Medical Centre, P. O. Box 3010, Kilimanjaro, Tanzania; 2 Kilimanjaro Christian Medical University College, P. O. Box 2240, Kilimanjaro, Tanzania

**Keywords:** Performance based financing, PBF pilot project, Rungwe, Tanzania

## Abstract

**Background:**

In recent years, Performance Based Financing (PBF); a form of result based financing, has attracted a global attention in health systems in developing countries. PBF promotes autonomous health facilities, motivates and introduces financial incentives to motivate health facilities and health workers to attain pre-determined targets. To achieve this, the Tanzanian government through the Christian Social Services Commission initiated a PBF pilot project in Rungwe district, Mbeya region. Kilimanjaro Christian Medical Center was given the role of training health workers on PBF principles in Rungwe. The aim of this study was to explore health care providers’ perception on a three years training on PBF principles in a PBF pilot project at Rungwe District in Mbeya, Tanzania.

**Methods:**

This was an explorative qualitative study, which took place at Rungwe PBF pilot area in October 2012. Twenty six (26) participants were purposively selected. Six took part in- depth interviews (IDIs) and twenty (20) in the group discussions. Both the IDIs and the GDs explored the perceived benefit and challenges of implementing PBF in their workplace. Data were manually analyzed using content analysis approach.

**Results:**

Overall informants had positive perspectives on PBF training. Most of the health facilities were able to implement some of the PBF concepts in their work places after the training, such as developing job descriptions for their staff, creating quarterly business plans for their facilities, costing for their services and entering service agreement with the government, improved record keeping, customer care and involving community as partners in running their facilities. The most common principle of paying individual performance bonuses was mentioned as a major challenge due to inadequate funding and poor design of Rungwe PBF pilot project.

**Conclusion:**

Despite poor design and inadequate funding, our findings have shown some promising results after PBF training in the study area. The findings have highlighted the potential of PBF to act as leverage for initiating innovative and proactive actions, which may motivate health personnel performance and quality of care in the study setting with minimal support. However, key policy issues at the national level should be addressed in order to exploit this opportunity.

## Background

The quality of health care services is deteriorating in many health facilities in low and middle income countries (LMIC) due to limited funds, limited management capacity and unmotivated health workers, [1-3].

Lack of resources for health care in LMICs; have prompted a renewed call for a focus beyond resource shortage to improvement in low motivation and poor performance among health personnel. In fact, currently particular attention is on the “know- do gap” – the gap between what health workers knowledge and what they actually do for their clients [3]. Performance-Based Financing (PBF) or Pay for Performance (P4P) has been touted as a promising strategy to address the aforementioned constraints.

Performance-Based Financing is a health system reform approach within the result based financing context, and defined as a cash or material goods transfer for measurable action in order to achieve predefined performance targets [4]. Performance based financing is directly linked to the principal - agent framework [5]; emphasizing a separation of functions of purchasing and providing health services [4,5]. In terms of the principal -agent framework, purchasing of health services involves three layers of actor which interact together: purchaser; provider and/or consumers. This framework uses financial incentives in form of performance reviews, to encourage providers to deliver health services more efficiently and of high quality [4,5] in order to meet national health strategies or targets. This framework is intricately linked with the “contract theory” or “incentive theory” whereby Health care is financially based on measurable results, specification of the contracted services, tendering processes, and procedures for monitoring and reviewing contract performance [6].

The ultimate goal of PBF is to improve service delivery and emphasize the key principles of autonomous and effective management of health facilities in decision making. These components include the hiring of staff, purchase of drugs, medical supplies and equipment and separation of functions within the health system [4-6]. Indeed, PBF approach motivates health staff because it encourages autonomy of health facilities, innovations, participatory planning and fair remuneration of staff through performance bonuses [4-6].

Studies on PBF have shown that its mechanisms, with incentives performance or results, to be promising in improving both health financing and quality of care [7]. A well-known and rigorously evaluated PBF programme in Rwanda, demonstrated that the PBF mechanisms helped to increase service delivery, quality of care, and improved health personnel performance [8]. Despite such promising results, there is limited uptake of the PBF approach in LMICs, and even less empirical evidence of its effectiveness [9].

The delivery of quality health care in Tanzania, like many LMICs, is challenged by insufficient funds for health care delivery, poor quality of health care, lack of both human and material resources, and lack of performance incentives, which negatively affect motivation of health personnel at all levels [9-17].

### Result based financing in a Tanzanian context

Results based financing was introduced into Tanzania in 2008 by development partners. In 2009 the Ministry of Health and Social Welfare (MoHSW) supported by development partners started a Pay for Performance (P4P) pilot project in one part of the country. Preliminary results from the P4P pilot project have shown some promising results [17,18]. However currently, there is no official health financing policy or guidelines on result-based financing, including PBF in Tanzania [12,19,20]. It is noteworthy to mention that there is still an ongoing debate among policy makers in Tanzania on whether P4P is synonymous to PBF or not, with others perceiving PBF as an extreme version of the new public management philosophy, which advocates for a series of changes in the organization and management of public sector, including health [21].

Despite the policy gap, the government through Christian Social Service Commission (CSSC) joined seven countries (Rwanda, Zambia, Burundi, DRC, Cameroon, Tanzania and Central African Republic), to implement a PBF pilot project at Rungwe district, Mbeya Region. One of the objectives of the PBF pilot project was to train health personnel on PBF principles. In order to achieve the above, one training institution in Tanzania, the Kilimanjaro Christian Medical Centre (KCMC) was identified to train health personnel in PBF principles for the period of three years (2010 to 2012). The main goal of the intervention was to improve the quality health service provision by motivating health workers through performance bonus, increased autonomy of the health facilities and encouraging the adoption of a Service Agreement (SA) between the health facility and the government.

### Overview of the training

The overall objective of the training was to improve general knowledge and skills on how to implement the PBF intervention. The participants included different cadres of staff working in Faith Based Organization (FBO) and government health care facilities in Rungwe District.

Sixty participants were trained between 2010 and 2012. Each batch had 20 participants who came from different health facilities in Rungwe District. A pre-defined selection criterion, set by the Rungwe PBF project coordinator, was used and included: gender, professional background, management position at health facility, and geographical location of a facility. The selection of trainee was done by the management of respective health facilities. The 10 day training, modified from a Rwandan curriculum, covered 10 topics and used adult learning principles, and involved interactive lectures, small group discussions, group presentations, questions and answer, role-plays and field visits. Both trainees and facilitator’s manuals were developed and used during the training.

Every morning evaluation of the previous days’ sessions was done. Summary scores (items were scored by individual participant by indicating “good”, “average” and “poor” for each item) obtained were discussed to foster course improvements. The evaluation focused on; session organization, appropriateness of the teaching; quality of materials provided; cooperation and sharing ideas; complimentary between theory and practice; facilitation methods and adequacy knowledge on PBF. At the end of the training, a final assessment was also done.

## Methods

### Study design

We conducted an explorative qualitative study using in-depth interviews with key informants and group discussions with health personnel who participated in PBF training.

### Study area

The study was conducted in October 2012 in Rungwe district in Mbeya Region, Tanzania. The district has 47 dispensaries out of which 7 are owned by FBOs; five health centres of which 2 are owned by the FBOs; three hospitals out of which two are owned by FBOs. In addition the district has 703 registered health staff who works in Government or FBOs owned health facilities. Of these, 275 (39%) health personnel work in the FBOs health facilities, and 428 (61%) work in the Government health facilities.

### Study population and sampling

The study population included members of staff working in health facilities and institutions that were involved in the PBF training. In total 26 respondents were purposively selected: namely six key informants who worked in health facilities had been trained on PBF. Four group discussions took place involving 20 participants who had not been trained but had been exposed to PBF concepts through their colleagues who took part in the first and second round of PBF training.

In-depth interviews were conducted with six key informants (three heads of the health facilities, one Medical officer, one facility health administrator, and one PBF project staff). The interviews focused on their experiences of implementing PBF in their places of work (Table 1).

**Table 1:** Characteristics of participants (n = 26)

**Characteristic**	**In-depth interview**		**Group discussion**	
	**Female (n = 0)**	**Male (n = 6)**	**Females (n = 8 )**	**Male ( n = 12 )**
Level of health facility				
Private Dispensary	N/A	1	1	1
Private Health Centre	N/A	2	1	1
Private Hospital	N/A	1	4	4
Public health facilities	N/A	1	1	5
Health institution	N/A	1	1	1
Attended PBF training				
Yes	N/A	6	8	12
No	N/A	0	0	0
Year of Training				
2010	N/A	1	0	0
2011	N/A	2	0	0
2012	N/A	N/A	8	12
Other*	0	3	0	0

*Trained on PBF outside the county (Zambia 2 and 1 Nairobi Kenya in 2009 and 2010 respectively).

### Data collection

There were two main interview guides, for in-depth interview (IDI) and group discussions. Both guides had four main sub-topics focusing on: (a) perceived benefits of the PBF training at individual level, (b) perceived benefits of the PBF training at their work place, (c) perceived challenges in implementing PBF, and (d) possible solutions to address the challenges. Key specific questions were: 1) in which ways was the PBF training beneficial to you and your health facility?; 2) In your opinion what effects or benefits had PBF training in your health facility? (Probe: planning, management, incentives, quality of care); and 3) could you describe the challenges which your facility faced in implementing PBF?

Interviews and group discussions were facilitated by the first author [RM] in Kiswahili-the official language of Tanzania and the group discussions by DM. Group members actively participated in the discussion. The discussions centered on design of the PBF pilot project at Rungwe, lack of performance bonuses and inadequate human resources in most of their facilities. The facilitators were both experienced in conducting qualitative studies. Both IDIs and group discussions lasted about sixty (60) minutes and were tape recorded. During the group discussions, notes were taken by an experienced recorder and expanded immediately after the discussions.

### Data management and analysis

All IDIs and group discussions were transcribed verbatim and translated into English independently by two researchers. The texts were read independently by researchers repeatedly to identify major themes. A framework approach guided the identification of key themes used to develop the thematic framework, which was systematically applied to all the data [22,23]. Translated transcripts were coded using words by the first author [RM], and verified by all co-authors. Discrepancies in coding were discussed among co-authors and a consensus was reached. Representative, verbatim quotes were selected to illustrate key findings.

### Ethical consideration

Ethical approval (No. 584; dated 2/10/2010), was obtained from Kilimanjaro Christian Medical University College Ethics review committee. All respondents consented to take part in the study and ethical procedures were adhered to.

## Results

The results of this study show that the trained participants returned to their work stations implemented the PBF approach to varying degrees of success. In this paper we discuss the findings under the following themes: perceived benefits of PBF training at individual and health facility levels and perceived challenges encountered during the implementation of PBF.

### Perceived benefits of PBF training at individual level

Overall informants had positive views towards the PBF approach and its benefits to them personally. These benefits included improvements to their management skills, their approaches to customer care and a newly acquired sense of responsibility in their work.

### Improved management skills and sense of responsibility

After attending the PBF training the participants returned and developed job descriptions for their staff. An in-charge of a private dispensary explained, “[…] *as a leader PBF training has made it possible for me to understand my responsibilities and I have been able to work with my staff to develop job descriptions for each staff; this has helped me in conducting supervision”* (Health facility in-charge IDI 1).

Key informants in the in-depth interviews were asked to mention the results of the PBF training. One hospital administrator explained:

*“PBF has changed me; I am no longer depending on any person to tell me what to do. I know my responsibilities and my employer is also satisfied […]”* He went on saying *“[…] the District Medical Officer (DMO) is appreciating our efforts to provide quality service; as a result, he has decided to second more staff in our hospital [….] we still have the human resource shortage but we are happy that somebody has noted our efforts of providing quality services”* (Hospital administrator).

The PBF approach encourages a degree of autonomy to allow service providers to be creative and innovative in order to improve the quality of care in their respective facilities. One such way is for each health facility to develop its own business plan stipulating activities to be achieved in each quarter. The facility business plan should be developed by all staff in participatory way in order to create a sense of ownership. Informants in both the IDIs and group discussions said that before PBF training they reported that they conducted their daily tasks without any business plan. After PBF training things changed in some of the facilities as narrated by a health facility in-charge, *“At first in our health facility, the in charge of the health facility was the only person responsible for planning […]. After the PBF training all staff are now involved in planning*. *We have developed a business plan which was not there. We have started to see some changes, we now have a monthly budget and the problem of drug being out of stock has been reduced to a great extent”* (Health facility in-charge IDI 2).

### Application of PBF principles and improved services at working place

Most of the benefits mentioned were those related to the workplace. Participants were able to apply modified PBF principles at no cost to achieve their goals. They grasped the concept of PBF as result based financing or output based financing. This is a shift from the input base financing approach, whereby there is redistribution of tasks and responsibilities between different actors. After the PBF training, performance bonuses were introduced using user fees in one of the government hospital as explained below:

*“Before I took part in the PBF training, our facility and health staff used to receive funding upon submission of report. After the PBF training, we modified this; we decided to pay staff after achieving specified targets such as certain percentage of immunization. Through this approach we have noted an increase of immunization coverage from 85% in 2009 to 95% in 2011. To us this is the success story”****(***Medical doctor in a government hospital; PBF project staff).

In addition to acquiring planning skills, informants also related PBF training with improved customer care as elaborated by in charge of a private dispensary, *“[…] knowledge of PBF has helped me to be more concerned and careful when I treat patients because if any thing goes wrong, they will not come back to us next time. I have also used negotiation skills gained during the training to enter into a contract with a company to get supply of laboratory items for our facility”* (Health facility in-charge IDI 1).

The availability of medical equipment is crucial to improve the quality of the service delivery to patients’. After conducting need assessment as part of PBF process, one of the facilities decided to buy an x-ray machine using funds from user fees as explained in the following narrative, “…*Our patients were walking too far for X-ray services. After the PBF training we decided to buy a new X-ray machine, this has attracted more patients in our hospital. Although we have no X-ray expert, wemanaged to negotiate with District Medical Officer (DMO) to allow an experienced person from the government hospital to come to work in our X-ray unit as per agreed schedule depending with the number of patients we have”* (Health facility in-charge IDI 3).

Across the groups, it was noted that, the staff that went through the PBF training developed some innovative ideas and became supportive to the management of their health facilities. During the interview with staff from government health hospital and during group discussions it was reported “[…] *staff that are trained on* PBF *are supportive, active and creative when we try to implement certain activities in the hospital. For example when we decided to pay staff after achieving specific outreach activities, others complained but we did not have any problem with those who attended PBF training”* (Medical officer in a government hospital; informants in group discussions 1 and 4).

### Improved financial management

Most of the participants cited that in their health facilities, there were no clear financial systems. After the PBF training, most of the in charge and administrator of health facilities sat together with other staff to establish financial system in their facilities. This resulted into positive views to the facility management, *“We like the financial transparency which we now see after our leaders attended the PBF training, all staff now know the income and expenditure of the health facility*” (Informants in group discussions 1 & 2). This was also narrated by an in-charge of a faith based private dispensary as follows, *“[…], formerly we had no good financial records, we now have our own bank account and some savings; we are freely using this money to buy medicine and other equipment without consulting the head office. Most important our books are annually checked by the Church auditor. We use part of the profit to motivate staff by giving them a token honorarium”* (Health facility in-charge IDI 3).

### Community involvement

Newly trained participants mentioned the establishment of health boards in their health facilities and community involvement as another benefit of PBF training. This was elaborated by in-charge of a private dispensary, “*We have been able to establish the governing board that includes the community members. The board chairperson is the village chief executive officer. This was possible only after PBF training we had. We were advised to form this board before even we were trained in PBF[…] I don’t know what prevented this to happen but after the training I gained an extra energy to follow up the matter with the Parish Priest who agreed and we started the board immediately”* (Health facility in-charge IDI 1).

All FBOs health facilities have a health facility committee, which work as gatekeeper of the health facilities and helping in the management of the facility. During the interviews and group discussions informants mentioned that some of the health facility committees have become more active. “*[…], following the PBF training, we decided to train the committee members on their roles and responsibilities using existing government guidelines. After the training, they [members], became more aware of their roles and started to be active in the management of our health facilities”* (PBF project staff).

Another result of training the health facility committee members was described as follows: *“[…]after establishing and training the facility health committees on principles of PBF and their roles, in one health facility they decided to do fundraising for getting medicine and medical supplies. Committee members invited people in the facilities and explained the real situation of why the facility is underperforming. They emphasized that the dispensary is theirs. As a result, people contributed”* (Hospital administrator; PBF project staff).

The PBF project coordinator met with health facilities board members to sensitize them on the PBF principles. This activity was appreciated by the health workers:

*“Board members do advise and assist in solving the health facility problems, for example, we had a critical shortage of staff, when we took the matter to the board meeting, the board made a follow –up with the DMO and some staffs were seconded to work in our hospital”* (Hospital administrator; PBF project staff).

One of the in charge of a health facility added that establishments of facility board has helped to improve communication between community and health facilities. He narrated:

*“The establishment of the board has helped us to have good communication with the community, for example, one of the board member informed us that women were afraid to give birth at our facility because they saw that every pregnant woman who comes in, immediately they hear the ambulance driving out with the pregnant woman to the referral hospital. We had to educate the board members about the risk factors of pregnancy. Later the board members educated the community on this issue and now we have seen an increase in the number of pregnant women who come to deliver at our facility. For example, from last January to September we had 49 deliveries. In the same period this year we have 64 deliveries”* (Health Facility in-charge IDI 1; PBF project staff).

### Improved costing of services

A hospital administrator explained how PBF training assisted the hospital management in costing of services. He explained that*, “Before PBF training we were operating without knowing the actual cost of our services. After the PBF training we started costing our services. We observed that we were over charging our services on some areas. For example, we were charging a cesarean section an amount of Tsh. 120,000/= (equivalent USD 75). After the costing exercise, the price dropped. It may look as a loss, but we have attracted more patients and the turnover is much higher per year”* (Hospital administrator).

### Other improvement in service provision after PBF training

Across group discussions and interviews, participants mentioned several interventions, which were implemented after the PBF training. According to the informants these resulted into a number of improvements in health care delivery. The following quotes support these claims:

*“After the training instead of using suggestion box, we decided to introduce client’s satisfaction survey. After analyzing the results we tried to expand and improve our services to include Immunization, laboratory, Voluntary Counseling and Testing (VCT) and Prevention of Mother to Child Transmission (PMTCT). This also resulted in the increase of utilization of services and improved client’s satisfaction. In addition, we have reached out and engaged the community through religious and public meetings” (*Health facility in-charge, IDI 2).

Similar strategy was used in another faculty where the manager said that, “[…] *because the suggestion box were not effective, we have introduced a new system of evaluating our services through client’s satisfaction survey which is administered by one or two members of the board asking clients about the services they receive from us*” (Health facility in-charge IDI 1; informants in group discussions, 3 & 4).

### Challenges encountered in implementing PBF

Informants pointed out key challenges related to implementation of PBF principles in Rungwe District. One of the key challenges mentioned by most informants involved the design of the PBF project. It was narrated by one of medical officer that, “*[…], the PBF project in Rungwe did not work as expected. This reflects the poor designing of this program”*. He went on saying “*It was designed without knowing how the health system in Tanzania works; it is not possible for the government to purchase and fund all the business plans from the health facilities through Service Agreement (SA)”.* An informant from the government hospital said, “[…], *we invited private health facilities to sign a contract with government through service agreement, but we couldn’t buy their business plans because the district does not have such budget item […]”* (Medical officer in a government hospital).

Informants in IDIs and participants in group discussions mentioned a counterproductive effect of improvement of quality of services. This was perceived as a potential barrier to SAs as narrated by a private hospital administrator, *“The contract [Service agreement] we entered with the government through SA was to attend 500 pregnant women and children. We have improved our services and we have managed to treat more and above the 500 by 200% […] the money we were paid is the one indicated in the contract only. This is very little money compared to what we have invested; it is very demotivating”* (Hospital administrator).

Another informant described the design as a ‘hybrid’ as none of the intended and expected financial incentives were provided to motivate the staff: *“Rungwe project is a hybrid […] that is, not designed to fit pure PBF principles. It has no money to buy indicators. Assumption was to use money from service agreement (SA) but this was not possible because the basket fund is based on ceiling funds allocated to the health facilities by the Ministry of Health”* (PBF project staff; Health facility In-charge IDI 1; informants in group discussion 2).

Another informant mentioned delay of funds after implementing the SA as a potential challenge. Informants recognized the difficulties of signing the SAs and the funding implications related to payments of pre-agreed indicators as explained in the narratives below:

*“[…], the budget takes a long time to be approved, […]our budget was approved in July while implementation started in January, this forces us to continue working in hard condition which may jeopardize the quality of services we are struggling to achieve”* (Hospital administrator; PBF project staff).

*“[…] we have no SA with the government. Pregnant women and children are supposed to be treated free, but we had to charge them little money amounting to Tsh. 5000/= (equivalent USD 3) for normal delivery, they get everything in the hospital. This is a challenge because it contradicting the country policy”* (Health facility in-charge, IDI 2).

Furthermore, there was lack of autonomy even at the District level as explained by one of the informant, *“The district is not autonomous […], we added some of our employee in the staff establishment at the district level […], this was ignored by the higher office at the Ministry of Health”* (Hospital administrator).

### In adequate human resource for health

Participants felt that most health facilities in the district have shortage of human resource of all cadres, both medical and non-medical; this has created heavy workload among the few existing staff, *“[…] the workload due to more patients who are coming to our facilities due to improved quality of care has increased, however, staffing and materials has remained the same […] copping with the increase number of client is a challenge to us”* (Hospital administrator; Health facility in-charge IDI 1 and 2). This resulted into low staff extrinsic motivation as they were working hard but could not get performance bonuses they expected, *“[…] we work very hard but no bonus as indicated in the PBF”* (Informants in all group discussions).

## Discussion

The aim of this study was to explore the health personnel perspectives on three years PBF training at Rungwe district. Our findings highlighted the potential to implement PBF, post training, despite several challenges in implementation. One of the key issues raised by most informants was lack of job description at their work place. However, hospital administrators were able to design job descriptions for all cadres after attending the PBF training. It is well documented that workers who are aware of their roles and responsibilities are more likely to perform their duties better and hence improve the overall quality of care [6,10,24].

In order for health facilities to function effectively and efficiently, it is imperative that health care providers regardless of the level of operation or ownership must have full autonomy of managing their health facilities. One of the fundamental tools advocated in PBF is for each health facility to design a business plan. Business plans in PBF, are defined as a quarterly work plans, which are submitted to fund holders to obtain a contract based on the performance in terms of output and quality [5,6]. Prior to PBF training in Rungwe, none of the health facilities had business plans in place, but used annual plans, derived from 5 years strategic plans. After the PBF training, most health facilities developed their quarterly business plans in a participatory manner [6].

On the other hand, most informants reported to have improved advocacy and lobbying skills for health promotion activities after PBF training. Lobbying and advocacy skills are important in sensitizing the surrounding communities of the health care services provided at a health facility and to involve communities as partners in running the facility. This is in line with PBF principles, which emphasizes strengthening of the consumer voice towards influencing quality and access to health services [21].

Most informants raised concerns of lack of financial transparency and accountability prior to the PBF training. The PBF approach addressed these concerns through the encouragement of well-established financial systems, including annual financial auditing [21].

In this study, participants highlighted the importance of involving members of the community in the management of health facilities through health committees/or governing boards. The strategy increased committee member’s sense of ownership and motivated them to engage in fund raising activities to support the health facilities. In addition, improvement in communication between the health facilities and the surrounding communities also improved through community involvement [11,12].

The overview of how to obtain customer satisfaction was introduced in the PBF training in order to change participant’s attitudes towards their clients. Several studies on health care seeking behaviors have documented that the poor attitude of health care providers towards their clients, is a key barrier to access for services [10-12,25]. In this study, seeking client’s level of satisfaction through exit interviews and suggestion box in most health care facilities in the study setting was attributed to PBF training.

Most participants’ concerns on the contractual agreements were rooted on the design of the current Service agreement (SA) in Tanzania. The current SA in Tanzania started in 2007, whereby the district council establishes local agreements with all private health facilities, including FBOs to provide a negotiated package of health care services [14,15,25-28].

In Rungwe district, the SAs were used as an entry point to implement PBF by buying the agreed indicators. However, one of the key issues raised related to SA was the discrepancy of cost per service, which was not representative of the real cost at the health facilities level. Another key issue, raised was delay of payments to FBOs by the district council; hence increased running costs of respective health facilities.

Some of the informants concerns on the contractual agreements may be an outcome of the PBF pilot project design. Contrary to a PBF pilot project implemented in Zambia by Churches Health Association of Zambia (CHAZ), the Rungwe PBF project was regarded as a “hybrid” PBF project. The key difference between the two PBF projects was that the Rungwe PBF project did not have funds to buy prior agreed indicators for performance and quality of care from business plans in respective health facilities, while CHAZ were provided with funds to buy for agreed indicators for services.

This mishap in the project design was fundamental in the implementation of Rungwe PBF project and highlighted the importance of incorporating all key PBF principles in the design of PBF projects in the future [6,15,21,29]. However, it is noteworthy to mention that performance incentives may not necessarily result in improved health outcomes, primarily because the incentive is related to effort and not outcomes [21,29]. In fact, a study done in Mvomero, Tanzania among health care providers involved in a P4P project, reported adverse effects, such as use of coercive strategies in order to reach set targets [30]. While proponents of performance strategies in health have argued that monetary incentives are most effective motivators, it is imperative to note that there can be “hidden-cost” , such as decreased intrinsic motivation in such rewarding systems [24]. Further studies in the context of motivation and financial incentive systems is warranted to assess the sustainability of such improvements observed among health personnel in the study area.

In this study, a number of challenges were noted during the implementation of the PBF project. The challenges raised by the participants have highlighted the importance of engaging key stakeholders in health during the design phase. For example, during the design stage of the Rungwe PBF project, it was assumed that funds from the District Health Administrator through SAs would be used to buy indicators for services, quality of care and performance bonuses. However, such activities are not allocated for in the current Comprehensive Council Health Plan guidelines in Tanzania [25]. The Rungwe PBF pilot project had funds for implementation of activities, but lacked funds to buy indicators. Secondly, lack of an official policy guideline on result-based financing, including PBF in the country may have a negative influence in the smooth implementation of the PBF project in Rungwe.

### Study limitations

This study has several limitations. First, the study was conducted by the same PBF training team; which may have contributed to social desirability bias, regarding informant’s responses on the success of the PBF training. However, the team tried as much as possible to be critical and objective. Second, the study lacks preliminary baseline information regarding the health personnel knowledge on PBF prior to the training and may have affected the study findings. Lastly, study informants were purposively selected and may not be representative of other individuals or other settings, hence lack external validity.

## Conclusion

In conclusion, our findings have shown promising results of PBF training to facilitate improvement in motivation and performance of health personnel in the study setting. The findings have highlighted the potential of training to act as leverage for initiating innovative and proactive actions, which may motivate health personnel performance and quality of care in the health facilities. Despite various pit-falls in the PBF project design, such as lack of performance bonus, health care personnel were motivated, and successfully implemented the PBF principles by exercising and extending their existing capacities to overcome some of the challenges. It is important that Tanzania as a country should develop a comprehensive health financing policy and guidelines on results-based financing, such as PBF, or P4P, through collaboration with all stakeholders in health. In addition, efforts should be made by both the government and private health providers to review the current Service Agreement template, so that is should not only focus on the judicial aspect of rigid contracts, but also incorporate supportive partnerships among stakeholders of health at all levels of health care delivery.

## Competing interests

The authors declare that they have no competing interests.

## Authors’ contributions

RM participated in the study design, data collection, and analysis and drafted this paper, DM participated in the study design and data collection, and data analysis, JK participated in the study design and data collection, SS participated in the study design and data collection, and BN participated in the study design, data collection and analysis. All authors participated in the writing of the manuscript and approved the final manuscript. All authors read and approved the final manuscript.

## Pre-publication history

The pre-publication history for this paper can be accessed here:

http://www.biomedcentral.com/1472-6963/14/154/prepub
